# Natural killer cells: the next wave in cancer immunotherapy

**DOI:** 10.3389/fimmu.2022.954804

**Published:** 2022-07-27

**Authors:** Xin Chen, Lei Jiang, Xuesong Liu

**Affiliations:** Department of Biology, BeiGene (Beijing) Co., Ltd., Beijing, China

**Keywords:** natural killer (NK) cells, immune checkpoint, cancer, immunotherapy, NK cell therapy, iPSC-NK

## Abstract

Immunotherapies focusing on rejuvenating T cell activities, like PD-1/PD-L1 and CTLA-4 blockade, have unprecedentedly revolutionized the landscape of cancer treatment. Yet a previously underexplored component of the immune system - natural killer (NK) cell, is coming to the forefront of immunotherapeutic attempts. In this review, we discuss the contributions of NK cells in the success of current immunotherapies, provide an overview of the current preclinical and clinical strategies at harnessing NK cells for cancer treatment, and highlight that NK cell-mediated therapies emerge as a major target in the next wave of cancer immunotherapy.

## Introduction

NK cells, a subset of lymphocytes that are principally innate immune cells, arise from common lymphoid progenitors and constitute the third lymphoid lineage in addition to T-cell and B-cell lineages (1). NK cells were initially discovered and named based on their ability to kill cancer cells *in vitro* (2). They express a broad repertoire of activating and inhibitory receptors, the “net weight” of which controls the final outputs. The biology of NK cells has been extensively reviewed elsewhere (3, 4). In this review, we mainly focus on the therapeutic potential of NK cells as the next wave in cancer immunity. We will discuss the prognostic roles of NK cells in cancers, summarize the contributions of NK cells in the success of immune checkpoint blockade (ICB) therapies and approaches including cell therapies to harness NK cells in the cancer treatment.

## NK cells in cancers

The immune surveillance role of NK cells in human cancers was first implicated in 1980s by reports revealing higher incidence of cancers in patients with NK cell defects (5, 6) and low NK cell activities in cancer patients or their families (7–11). Subsequently, a landmark 11-year following-up study reported a positive correlation between impaired NK cell functions and higher risk to develop numerous types of cancers (12). Meanwhile, the critical role of NK cells in control of tumor growth and metastasis was demonstrated in mice models in early studies (13, 14). However, due to the paucity of NK cells usually overserved in primary tumors in clinic, questions have been raised – as to whether NK cells play an important role in tumor control and prognosis, and whether NK cells contribute to therapies such as targeted antibody therapies, despite the role of NK cells in immune surveillance.

Subsequent to early findings, accumulating evidence have reported impaired functions of NK cells in chronic myelogenous leukemia (CML) (15) and acute myeloid leukemia (AML) (16, 17). Intriguingly, NK cells in AML patients have been reported to significantly down-regulate activating receptor NKp46 and up-regulate inhibitory receptor NKG2A compared to those in healthy age-matched controls (17). Furthermore, lower NKp46 expression on NK cells (18), phenotypic and functional defects of NK cells (17) or defective NK cell maturation (19) have been reported to be associated with adverse clinical outcomes in AML patients treated with allogeneic stem cell transplantation (allo-SCT) (18) or chemotherapy (17, 19).

Furthermore, the prognostic role of NK cells has not only been observed in chemotherapy-based studies in hematopoietic cancers, but also observed in targeted antibody therapy-based studies, in both liquid and solid tumors (**Table 1**). In diffuse large B-cell lymphoma (DLBCL) patients treated with Rituximab-CHOP (20), breast cancer patients treated with anti-HER2 monoclonal antibody (mAb) and chemotherapy (22), and in colorectal cancer patients treated with anti-EGFR mAb and chemotherapy (24), the tumor-infiltration of NK cells have been reported to positively correlate with clinical responses. Moreover, high baseline of antibody-dependent cellular cytotoxicity (ADCC) has been reported to correlate with a complete response (CR) and a long overall survival (OS) in head and neck cancer patients treated with anti-EGFR mAb and radiotherapy (23). Those evidence suggested a role of NK cells in targeted antibody therapy, probably mediated by ADCC, and support the development of tools harnessing ADCC activities of NK cells for enhanced anti-tumor efficacy. We will expand the discussion in later sessions of the review.

**Table 1:** Clinical correlations of NK cells with patient outcomes.

Cancer type	Treatments	Correlation of NK phenotypes with clinical outcomes	References
AML	Chemotherapy	Phenotypic and functional defects of NK cells associate with poor response.	(17)
	Conventional chemotherapy with or without the addition of anti-CD33 mAb	Patients with hypomaturation profile had reduced OS and progression-free survival (PFS) rates.	(19)
	Allo-SCT	NKp46^high^ phenotype at diagnosis is associated with better PFS and OS.	(18)
DLBCL	Rituximab-CHOP (cyclophosphamide, doxorubicin, vincristine, and prednisone)	Lack of NK cell infiltration associate with poor survival.	(20)
CML	Imatinib	NK cell counts are associated with molecular relapse-free survival after imatinib discontinuation.	(21)
Breast cancer	All patients received a neoadjuvant combination treatment of standard chemotherapy and anti-HER2 mAbs	Tumor-infiltrating NK cells associate with pathological CR and disease-free survival.	(22)
Head and neck cancer	anti-EGFR and radiotherapy	High baseline of ADCC correlates with a CR and a long OS.	(23)
Colorectal cancer	A first-line anti-EGFR based chemotherapy	Tumor infiltrating CD56^+^ cells are correlated with PFS and response.	(24)
Melanoma	Anti-PD-1 mAbs	Higher NK cell infiltration in responding *vs* non-responding patients.	(25)
	Anti-PD-1 mAbs	Up-regulated NK signatures and higher NK cells infiltration in tumors in responding *vs* non-responding patients.	(26)

Another intriguing observation related to the prognostic and predictive role of NK cells comes from the studies on immune checkpoint blockades (ICBs) therapies. Higher NK cell infiltration has been found in responders to anti-PD-1 treatment compared to non-responders from independent studies (25, 26), and thus raise the question whether NK cells contribute to the success of ICBs.

## NK cells contribute to the success of ICBs

Many inhibitory receptors including PD-1, LAG3, TIM3, TIGIT, NKG2A etc. are expressed and mediate inhibition on both NK cells and T cells (27) (**Table 2**). To date, anti-PD-1/PD-L1 therapies have achieved remarkable efficacy in a wide spectrum of cancers (28). Moreover, ICBs targeting LAG3 (29) and TIGIT (30) are displaying great potentials to further improve clinical outcomes in combination with anti-PD-1 therapy. Basically, the efficacy has been attributed to unleashing T cell responses, leaving the contributions of NK cells yet to be fully explored. Recently, growing evidence is suggesting a prognostic role of NK cell activation status and tumor infiltration in the success of ICB (25, 26, 31), thus raising considerable interests to fill the conceptual gap with respect to whether and how NK cells play a role in the ICB practice.

**Table 2:** Selected shared immune checkpoint receptors between NK cells and T cells.

Receptor	Cell distribution	Drugs approved or in advanced clinical trials	Phase
PD-1	NK cells, T cells, B cells, myeloid cells	Pembrolizumab	FDA Approved
		Nivolumab	FDA Approved
		Cemiplimab	FDA Approved
		Dostarlimab	FDA Approved
		Tislelizumab	Phase III in US; approved in China
TIGIT	NK cells, T cells	Tiragolumab	Phase III
		Vibostolimab	Phase III
		Ociperlimab	Phase III
TIM3	NK cells, T cells, DCs, monocytes, macrophages, mast cells	MBG453	Phase II
		BGB-A425	Phase I/II
		TSR-022	Phase II
NKG2A	NK cells and T cells	Monalizumab	Phase III

First, NK cells may contribute to the ICB success by restraining the emergence of cancer cell clones that have escaped T cell attack through inactivation of antigen presentation. There is growing evidence that loss of genes associated with antigen presentation serves as an important mechanism of acquired resistance to ICB (32). In pre-clinical models, Nicolai et al. and Das et al. showed that NK cells mediate the rejection of CD8^+^ T cell resistant B2m^-/-^ tumors (33, 34). It is in line with the longstanding observations that NK cells express inhibitory receptors binding to MHC-I, thereby maintaining “self-tolerance” to normal cells. When cancers down-regulate MHC-I on their surface to escape T cell attack, the “missing-self recognition” by NK cells is triggered, thus initiating NK cell mediated cytotoxicity against the “escapers” (35).

Second, the ICB may confer a direct modulation on NK cell activity. One study reported that PD-1 is upregulated on circulating and intra-tumoral NK cells in patients of Hodgkin lymphoma. PD-L1^+^ myeloid cells efficiently suppress the function of PD-1^+^ NK cells *in vitro*, while anti-PD-1 treatment can effectively reverse the suppression (36). Further evidence for the PD-1/PD-L1 signaling in NK cells comes from studies describing PD-1 upregulation in NK cells in non-small cell lung cancer (NSCLC) and head and neck cancer (HNC) patients. PD-L1 beads or PD-L1^+^ target cells impaired PD-1^+^ NK cell function, while anti-PD1 or anti-PD-L1 treatment significantly activated PD-1^+^ NK cells *in vitro* (37, 38). Moreover, there is evidence in *in vivo* mouse models that PD-1 is up-regulated on most activated tumor-infiltrating NK cells, and NK cells mediate full therapeutic efficacy of PD-1/PD-L1 blockade (39). Nevertheless, to what extent the anti-PD-1/PD-L1 therapies could directly activate NK cells in patients and thereby contribute to the efficacy remains an open question that needs to be further explored. Another shared checkpoint between T cells and NK cells, TIGIT, is constitutively expressed on PBMC-derived NK cells as well as *in vitro* activated human NK cells (40–43). In a recent publication, we have demonstrated the direct activation of NK cells by the therapeutic TIGIT blocking antibody ociperlimab (BGB-A1217) in an *in vitro* NK-cancer cell co-culture assay (44). Remarkably, the full Fc effector function of ociperlimab further elevated NK cell function in addition to checkpoint blockade (44), probably through the synergy between FcγRIIIa (CD16a) signaling and release of TIGIT mediated suppression on NK cells (45). Another immune checkpoint, TIM3, has been found to be up-regulated on NK cells from patients with melanoma (46), gastric cancer (47) and lung adenocarcinoma (48), and blockade of TIM3 has been reported to release the exhaustion of NK cells from advanced melanoma patients *in vitro (* 46).

Recently, the NKG2A/CD94 blockade seems to carve a new path in the adoption of ICB in the cancer treatment *via* unleashing both T cells and NK cells. Pre-clinical data suggest a dual role of NKG2A blockade on NK cells and T cells (49–51). In clinic, monalizumab (49), a humanized IgG4 ICB targeting the NKG2A/CD94 receptor, blocking its interaction with HLA-E, is being investigated in the treatment of solid tumors. Encouraging results from a large, randomized Phase II trial showed monalizumab in combination with durvalumab, a PD-L1 blockade antibody, improved PFS and objective response rate (ORR) compared to durvalumab alone in patients with unresectable, stage III NSCLC. The 12-month PFS rate was 72.7% for durvalumab plus monalizumab, versus 33.9% with durvalumab alone (52).

From another perspective, it is noteworthy that the immune checkpoint blockade antibodies can activate NK cells through the Fc effector functions, as we reviewed previously (53). Direct evidence for this hypothesis comes from the Fc-competent TIGIT antibody Ociperlimab. TIGIT expression is highly expressed on Treg cells, relative to effector T cells, and is further elevated on Tregs in tumor microenvironment (44, 54). Our data have shown that the ligation of TIGIT on Tregs and Fcγ receptors on NK cells by Ociperlimab directly promoted NK cell activation and induced ADCC against cancer patient PBMC derived Tregs *in vitro*. In the CT26 mouse model, we also observed the decrease of intratumor Treg numbers (44). It is of great interest to further explore the potential mechanisms in clinical settings. Another T cell checkpoint, CTLA-4, is also expressed on cancer cells such as melanoma, leading to potential NK cell mediated ADCC against CTLA-4^+^ cancers induced by anti-CTLA-4 treatment (55). Nevertheless, CTLA-4 is also expressed on CTLs, thus rendering the overall mechanisms complicated.

Third, emerging evidence have suggested an essential role of NK cells in checkpoint therapy response through an NK-dendritic cell (DC) axis (25, 56). Conventional type 1 dendritic cells (cDC1) are a subtype of DC that stimulate robust T cell response to cancer. They adept at taking up dead cells and cross-present tumor antigen to CD8^+^ T cells (57, 58), attract T cells into tumor (59), and elicit tumor-specific T cell responses (60). Intriguingly, work from Bottcher et al. revealed a strong correlation between cDC1 signatures and NK cell signatures in cancers including skin cutaneous melanoma (SKCM), breast invasive carcinoma (BRCA), head and neck squamous cell carcinoma (HNSC) and lung adenocarcinoma (LUAD) (56). Moreover, NK cell signatures were found positively associated with patient survivals in all those cancers. Furthermore, they discovered in pre-clinical models that intratumor NK cells recruit cDC1 into tumors to promote tumor control (56). Similarly, Barry et al. observed that NK cell signatures positively correlate with stimulatory dendritic cells (SDC; intratumor cDC1) in melanoma, patient response to anti-PD-1 therapy and overall survival. In line with the data from Bottcher et al., they also uncovered a role of NK cells in the control of CD103^+^ SDC in a mouse tumor model (25).

Taken together, NK cells may contribute to ICBs success through multiple aspects. However, one caveat is that one should be cautious to interpret the data from pre-clinical models and translate from laboratory to clinic. Gaps exist between mouse models and human cancers, e.g., FcγRIII on mouse NK cells is actually not the homologue of FcγRIII on human NK cells. Human FcγRIIIa is functionally similar with a unique mouse FcγR – FcγRIV (61). Nonetheless mouse FcγRIV is not expressed on mouse NK cells, but abundantly on macrophages (53). In addition, syngeneic or xenograft tumor models may not truly mimic the NK cell infiltration status in human tumors, thereby suggesting translational gaps between pre-clinical tumor models and cancer patients.

## Approaches to harness NK cells

NK cells express a broad range of activating and inhibitory receptors. Whether NK cells attack a target cell depends on the net equilibrium of the activating and inhibitory signals. Here we focus on emerging novel modalities for NK cell targeting, e.g., ADCC enhanced antibodies, bi- or tri-specifics, and iPSC-derived NK cells (iPSC-NK) therapies.

### ADCC-enhanced antibodies

In humans, FcγRIIIa is the major type of FcγRs expressed on NK cells (62). Binding of Fc portion of human IgG to FcγRIIIa can trigger NK cell ADCC against mAb-opsonized target cells, as has been firmly established. Two alleles encode different FcγRIIIa variants that differ at the position 158, with either a valine (V) or phenylalanine (F). Between the two isoforms, FcγRIIIa-V_158_ exhibits higher affinity to IgG1, and mediated more efficient ADCC (63). In clinic, the FcγRIIIa dimorphism was strongly associated with the outcome of patients treated with anti-EGFR or anti-CD20 antibodies (64–69). Although it remains controversial about the relative contributions of different immune cells or effectors in the therapeutic efficacy of tumor-targeting mAbs (70–73), multiple studies have suggested a positive correlation of NK cell infiltration and activity with the response to tumor-targeting mAb treatment (22–24), and again, caution is warranted on the interpretation of mechanistic studies in mice given the discrepancies of FcγRs expression profiles between human and mice. Therefore, several strategies have been employed to develop ADCC-enhanced mAbs for harnessing NK cell functions.

Removal of core fucose from N-glycans attached to human IgG1 significantly enhances the binding affinity of IgG1 to FcγRIIIa and ADCC (74, 75), and has been the most widely adopted approach to harness the mAb mediated ADCC response in clinical practice (76). As of today, three afucosylated mAbs have been marketed for the treatment of human cancers: Obinutuzumab, a CD20-directed afucosylated antibody approved for the treatment of chronic lymphocytic leukemia (CLL); Poteligeo (mogamulizumab), a CCR4-targeting afucosylated mAb, approved for the treatment of Mycosis Fungoides (MF) and Sézary Syndrome (SS); and Fasenra (benralizumab), an afucosylated IL-5Rα targeting mAb for the treatment of patients with severe eosinophilic asthma. In addition, Rybrevant (amivantamab), an anti-EGFR and anti-cMet bispecific low fucose antibody with enhanced Fc function, have been approved for the treatment of NSCLC. Blenrep (belantamab mafodotin-blmf), consisting of an afucosylated humanized anti-BCMA IgG1 mAb conjugated to the tubulin inhibitor, monomethyl auristatin F (MMAF), for the treatment of adult patients with relapsed or refractory multiple myeloma, is the only FDA approved ADC with an afucosylated antibody (77). Nowadays, numerous afucosylated mAbs targeting a diverge range of receptors are actively in clinical development, with the outcomes yet to be revealed (78).

Fc engineering represents another approach to enhance ADCC (62, 79). Several Fc-enhanced mAbs through the genetic engineering approach are being investigated in clinical trials, with only one approved by FDA till now, Margenza (margetuximab), for the treatment of metastatic HER2-positive breast cancer. It is noteworthy that exploratory PFS analysis by FcγRIIIa genotype suggested that presence of a FcγRIIIa-F_158_ allele may predict margetuximab benefit over trastuzumab. Margetuximab provided no clinical benefit in FcγRIIIa-V_158_ homozygotes compared with trastuzumab (80). Since the Fc engineering of margetuximab-cmkb increases affinity for both FcγRIIIa allotypes, and FcγRIIIa-V_158_ per se has higher affinity to IgG1, the none-benefit in FcγRIIIa-V_158_ homozygotes might be attributed to the rapid cleavage and downregulation of FcγRIIIa due to stronger binding of the antibodies to FcγRIIIa-V_158_. From another perspective, strong binding to FcγRIIIa may induce enhanced antibody internalization by FcγRIIIa expressing cells, thus promoting the anti-drug antibody (ADA) production, to compromise the efficacy. The exact underlying mechanisms are yet to be elucidated.

### NK cell engagers

There are a broad range of activating and inhibitory receptors on NK cells. The integration of signals for activation and inhibition determines the final outputs of NK cells. The loss of inhibitory signaling, like downregulation of MHC-I expression on tumor cells, renders tumor cells susceptible to NK cell cytotoxicity. Alternatively, NK cells can attack cancer cells that retain full expression of MHC-I if activating receptors on NK cells are engaged.

Recently, bi-specific or tri-specific antibodies targeting NK cell activating receptors are emerging as novel approaches to harness NK activity. Preclinical results provide the rationale for developing multi-specific NK cell engagers through ligation of tumor antigens and activating NK receptors. Examples include those targeting NKp46 (81), NKp30 (82), NKG2D (83, 84), and FcγRIIIa (CD16a) (85, 86). Encouraging data comes from a Phase I clinical study in which an anti-CD16/anti-CD30 bispecific NK-cell engager combined with pembrolizumab has shown an ORR of 83% and a CR rate of 46% in patients with relapsed or refractory Hodgkin Lymphoma (HL) (87). However, it should be noted that most of the activating receptors are not exclusively expressed on NK cells, instead are often shared with T cells or myeloid compartments. Targeting the activating receptors on NK cells may synergically augment both NK cells and other immune effectors.

### NK cell-checkpoint blockades

A wide range of immune checkpoints are expressed on NK cells. As with the activating receptors, NK checkpoints are usually shared with other immune components (27). As discussed in the earlier part of the review, some blockades that can target checkpoints on both T cell and NK cells have obtained remarkable success or promising preliminary clinical responses (**Table 2**), albeit the contributions of NK cells therein are yet to be fully understood. In addition, blockers that target inhibitory killer Ig-like receptors (KIRs) have been investigated in clinical settings. KIRs are a group of receptors on NK cells that bind to HLA molecules to mediate inhibitory or activating signaling (88). Clinical evidence have suggested that adoptive NK cell transfer has the potential to improve outcomes of KIR ligand-mismatched recipients even further (89–91). Lirilumab, a humanized IgG4 mAb, binds to KIR2DL-1, KIR2DL-2 and KIR2DL-3 and thereby blocks their inhibitory signaling mediated by both HLA-C C1 and HLA-C C2 subtype molecules (92). It has shown good safety profiles in phase I trials, however, the phase II trial in patients with smoldering multiple myeloma failed to demonstrate clinical efficacy (93). The minimal efficacy may result from lack of the KIR matched HLA types from patients, and existing of other dominant inhibition signals (94). However, this does not rule out the possibility that inhibitory KIR blockers could synergistically work together with other ICBs or NK cell therapies to induce a combination efficacy.

### NK cell-based cell therapies

Adoptive cell therapies, basically chimeric antigen receptor T (CAR-T) cell therapies, have exhibited remarkable clinical responses in treating hematologic malignancies, and thus spawned an explosion in the CAR-T field. As of today, six CAR-T cell therapies have been approved by FDA, wherein four targeting CD19, and two targeting BCMA. Although the efficacies have been notable (95–101), limitations are still obvious. First, as a highly personalized therapy, autologous CAR-T cells have to be individually prepared for each patient in a time- and material- consuming process that carries the risk for failure and demanding logistics. Patients who have already received multiple rounds of chemotherapy may not be able to mobilize enough T cells for the CAR-T cell preparation. Additionally, during the time waiting for CAR-T cells manufacturing, patients may experience disease progression. As such, the therapy results in low scalability and remains unacceptable and unaffordable to most of patients. Second, severe toxicity associated with CAR-T cells hampers the broad applicability of the treatment. In several patients, CAR-T cell treatments have been associated with substantial toxicity including cytokine release syndrome (CRS) and immune effector cell-associated neurotoxicity syndrome (ICANS), thus decreasing the feasibility due to demanding toxicity management, and inconvenient administration (102).

In contrast, NK cell therapies harbor high potential to overcome those hurdles. Firstly, the activating machineries of NK cells differ from the TCR system of T cells. NK cells do not require HLA matching to exert cytotoxicity against tumor cells. This allows for using NK cells in an allogeneic way. On the other hand, allogeneic NK cells do not result in graft versus host disease (GvHD), even in the setting of substantial HLA disparity between adoptive CAR-NK cells and the recipients (103), and thus can be provided as complete “off-the-shelf” products, significantly lowering the cost of manufacturing and logistics. Secondly, both autologous and allogeneic NK cells have exhibited excellent safety profile, without severe toxicity such as CRS or ICANS (103, 104). Compared to their T cell counterparts, NK cells present a safer cytokine profile, and differ in the crosstalk with myeloid cells (4). This property confers the feasibility of the NK cell therapy when specialized care units are unavailable. Thirdly, from the efficacy perspective, NK or CAR-NK cell therapies have shown inspiring clinical outcomes in early phase clinical trials when used alone (103) or in combination with other therapies (104), encouraging more endeavors in the field. Last but not least, repeated doses can be administrated given the short lifetime of NK cells, and NK cells from different donors can be sequentially dosed to circumvent rejection of donor NK cells by recipient memory T cells recognizing allo-antigens on the same donors.

NK cells for cell therapy can be generated from different sources and by a variety of methods (**Table 3**). Peripheral blood derived, and ex vivo expanded autologous NK cells have been well tolerated in clinical trials, whereas efficacy has been limited (105, 106). The low efficacy may be attributed to the suppression of autologous NK cells by self-HLA molecules. As such, allogeneic NK cells serve as a promising alternative approach to overcome the resistance. In a seminal study by Miller et al., a complete remission induced by haploidentical allogeneic NK-cell infusions in 5 of 19 poor-prognosis AML patients was observed (90). In a subsequent study in pediatric AML, all patients who received adoptive haploidentical NK cells remained in remission with a median follow-up time of 964 days (91). Later on, 53% complete remission was observed in AML patients treated with haploidentical NK cells combined with an IL-2 diphtheria toxin fusion protein, which was used to deplete host regulatory T cells (107); 32% complete remission was observed in AML patients treated with haploidentical NK cells combined with IL-15 (108), and 44% complete remission was observed in AML patients treated with allogeneic cytokine-induced memory-like NK cells, in separate studies (109).

**Table 3:** Comparison of clinical-scale NK cells generated from distinct sources.

Attributes	NK-92	PB-NK	UCB-NK	HPC-NK	iPSC-NK
Source	NK-92 cell line	Peripheral blood of donors	Cryopreserved umbilical cord blood (UCB) in UCB unit	CD34^+^ hematopoietic progenitor cells from UCB	iPSC
Tumorigenicity	High (need to be irradiated before infusion)	Low	Low	Low	Low
Accessibility	Easy	Easy	Less easy	Less easy	Easy
Homogeneity	High	Low	Low	Low	High
Genetic engineering	Easy	Less easy	Less easy	Less easy	Easy
Cell number sufficiency of a uniform cell population for repeated doses	High	Low	Low	Low	High

Besides peripheral blood-derived NK cells (PB-NK), an alternative approach to generate functional NK cells is to obtain NK cells from umbilical cord blood and expand *ex vivo* (110, 111). A recent clinical study by Liu et al. has presented inspiring results to show the remarkable efficacy and excellent safety profile of engineered umbilical cord blood derived NK cells (UCB-NK) in the treatment of CD19 positive relapsed or refractory lymphoid tumors. The HLA-mismatched UCB-NK cells were transduced with a retroviral vector encoding anti-CD19 CAR, IL-15, and inducible caspase 9 as a safety switch. Of the 11 patients who were treated, 8 (73%) had a response and 7 (64%) had a complete remission. Notably, no severe toxicity including CRS, neurotoxicity or GvHD were observed (103). Another method to derive NK cells from umbilical cord blood is to differentiate them from CD34^+^ hematopoietic progenitor cells (HPC) (112). In the first-in-human study, CD34^+^ HPC derived NK cells (HPC-NK) were administrated to 10 older AML patients after lymphodepleting chemotherapy without cytokine boosting. Preliminary data showed that HPC-NK cells were well tolerated, with neither GvHD nor toxicity observed. Notably, 2 of 4 patients with minimal residual disease (MRD) in bone marrow before HPC-NK cells infusion became MRD negative, which lasted for 6 months (113).

Albeit the encouraging efficacy achieved by those clinical trials using PB-NK or UCB-NK, limitations exist due to the requirement for collection from a donor by apheresis or from umbilical cord blood, the variability of NK cell yield influenced by donor variability, and the challenge in generic manipulation on differentiated cells with low proliferation capacity. To overcome those limitations, a number of studies used NK-92, a NK cell line originally established from a patient with non-Hodgkin’s lymphoma (114–116). There are several advantages of NK-92 cell line as a source for NK cell therapy – it provides a homogeneous master cell bank, can be expanded indefinitely and served as an uniform “off-the-shelf” product, is more amenable to genetic modification and allows sufficient cells for cell therapy (117). However, on the other hand, albeit NK-92 lack expression of most known KIRs and exhibit broad cytotoxicity against numerous cancers, it loses expression of typical activating receptors including NKp44, NKp46 and notably, FcγRIIIa, which mediated ADCC (117). Additionally, as a lymphoma cell line, NK-92 holds inherent draw backs such as potential tumorigenicity and latent infection by Epstein-Barr Virus (EBV). Thus, for safety considerations, NK-92 must be irradiated before administration to patients. The irradiation limits the proliferation and persistence of NK-92 *in vivo*, and eventually may impede the long-term anti-tumor efficacy. And this may account for the observed limited efficacy of NK-92 cells in clinic (118, 119).

In recent years, iPSC-NK technology emerges as a breakthrough innovation in the NK cell therapy field, offering the potential to overcome challenges often seen with other source-derived NK cells. Serial seminal studies from Kaufman et al. have significantly optimized the protocols to derive NK cells to a clinical-scale from embryonic stem cells (hESCs) or iPSC (120), and demonstrated for the first time that CAR-NK cells can be derived from iPSCs expressing CAR (121). Since pluripotent stem cells have the potential to grow indefinitely in an undifferenced state (122, 123), the iPSC can serve as a stable cell bank for uniform NK cell generation and allows for sufficient cell numbers for cell therapy. As such, the iPSC-NK can serve as a standardized “off-the-shelf” product. In addition, iPSC is amenable to genetic engineering. Once the genetically modified clones are selected, it can be expanded for a production of a uniform pool of iPSC-NK cells. Multiple genetical modifications, such as ectopic expression of IL-15/IL-15R fusion protein (124, 125), CAR (121), high-affinity non-cleavable variant of CD16a (125, 126), deletion of CISH (127) or CD38 (125) have been successfully introduced on iPSC-NK to achieve enhanced expansion, better *in vivo* persistence or greater cytotoxicity. The difference between iPSC-NK, PB-NK and UCB-NK are yet to be fully understood, yet some pre-clinical evidence have suggested that iPSC-NK may have comparable or superior activities relative to PB-NK or UCB-NK (128–130). To date, iPSC-NK cell therapies have entered phase I clinical trials, used alone or in combination with therapeutic monoclonal antibodies (mAbs) for the treatment of hematopoietic lymphomas or solid tumors (131–136). Remarkably, the first in human results are encouraging (137, 138). In a phase I trial, FT516, an iPSC-NK cell therapy using iPSC-NK cells engineered with a high-affinity, non-cleavable CD16a (hnCD16) that enables tumor targeting and enhanced ADCC in combination with a therapeutic mAb, was combined with rituximab to treat patients with relapsed or refractory B-cell lymphoma (BCL) (132). Eight of the 11 pts (73%) treated with ≥90 million FT516 cells achieved an objective response. Seven (64%) patients achieved CR, including 2 patients with prior CD19 CAR T-cell therapy (139). FT596 (140), is an iPSC-derived CAR-NK cell therapy armed with three modalities: a CD19-targeting CAR, a hnCD16, and IL15/IL-15 receptor fusion which promotes NK cell persistence by the autonomous cytokine. In a phase I trial, FT596 was administrated as monotherapy or in combination with rituximab or obinutuzumab for the treatment of relapsed or refractory BCLs and CLL (141). At single-dose levels of ≥90 million cells, 8 of 11 (73%) efficacy-evaluable patients achieved ORR, including 7 (64%) CR. Of 4 patients with prior CAR T-cell therapy treated at ≥90 million cells, 2 achieved CR (142). Notably, no dose-limiting toxicities, CRS, ICANS, or GvHD of any grade were observed for FT516 or FT596, and repeated doses were allowed (139, 142).

### NK cell-based combination strategies

Nowadays, NK cell-based combination strategies have been investigated in the cancer immunotherapy and represent an important direction in the future.

#### NK cell therapies in combination with ICBs

As we have discussed in the previous sessions, activated or intra-tumor NK cells up-regulate checkpoint molecules (e.g., PD-1, TIGIT, TIM3, NKG2A) and blocked of those molecules unleash NK cell activity. In a pre-clinical model, iPSC-NK cells in combination with T cells and an anti-PD-1 antibody have been reported to eliminate tumors in a xenograft ovarian cancer mouse model (143). Combination of ICBs and adoptive NK cell therapy would be a promising approach to achieve optimized NK functions, and in concert with T cells.

#### NK cell therapies in combination with tumor targeting mAbs or NK cell engagers

Adoptive NK cell therapy in combination with tumor-targeting mAbs or other NK cell engagers represent an approach to fully augment the tumor-specific NK cell cytotoxicity. Impressive results have been obtained from a phase I clinical study that combine CAR-iPSC-NK cell therapy with anti-CD20 (139). Moreover, the combination of CB-NK and a bispecific CD30/CD16 antibody is being actively investigated in a phase I/II study (144, 145). As disclosed by Affimed on the 2022 AACR meeting, as of the cut-off date, the study had enrolled 22 patients with relapsed or refractory CD30^+^ Hodgkin and non-Hodgkin lymphoma having received a median of seven prior lines of therapy. Out of the 13 patients treated at the recommended phase 2 dose (RP2D), 13 patients (100%) achieved objective response, and 8 (62%) patients achieved CR after two cycles of treatment (146).

#### CAR-NK and CAR-T cell combinations

Sequential infusions of CAR-NK cells and CAR-T cells would be a good strategy to achieve better efficacy and safety. CAR-NK cells should rapidly decrease the tumor burden, particularly for patients with high tumor load. This may decrease the CRS and neurotoxicity risk imposed by CAR-T cells. Then subsequent CAR T cell infusion may eliminate residual tumor cells and provide a lasting anti-tumor effect through memory T cells that survive and persistence.

### Other attempts to target NK cells

In addition to the approaches discussed above, strategies targeting cytokines, such as IL-12, IL-15 and IL18 (109, 124, 147), targeting intracellular checkpoints, such as CISH (127, 148), Cbl-b (149) GSK3 (150) and CDK8 (151, 152), or targeting tumor cells which can indirectly trigger NK cell surveillance through non–cell autonomous mechanisms (153) may also effectively augment NK cell functions and eventually result in novel therapeutic candidates.

## Conclusions and prospects

Taken together, NK cell-based therapies have attracted intense interest and shown great potential in the treatment of cancers, emerging as the next wave in cancer immunotherapy. Multiple approaches, including checkpoint blockades, ADCC enhanced antibodies, agonist antibodies and multi-specific NK cell engagers, and adoptive NK cell therapies (particularly engineered iPSC NK cell therapies) have significantly widened the pool of potential clinical options. However, challenges exist with the opportunities. As a heterogenous population, NK cells are still not fully understood. It is crucial to continue to delineate the NK cell biology and characterize the differences of NK cells derived from distinct sources and methods. In addition, although NK cell-based therapies have demonstrated great potentials in the treatment of hematopoietic cancers, the advances in solid tumors remain limited. It is important to further understand NK homing capacities and the reasons underlying their poor infiltrations in solid tumors, which may eventually lead to the development of novel approaches to overcome the barriers. Furthermore, the questions about the persistence of NK cells, and the durability of the response, and the affordable cost for patients need to be considered. Along with the advancing of new technologies and methods, NK cell-based therapies will continue to evolve, and get closer to benefit patients with otherwise no treatment options. In summary, NK cell-mediated therapies have emerged as the next wave in cancer immunotherapy.

## Author contributions

XC and LJ drafted the manuscript. XL reviewed the manuscript, and all authors were involved in revision of the manuscript. All authors contributed to the article and approved the submitted version.

## Funding

This work was supported by internal company funding.

## Conflict of interest

XC is a Senior Principal Investigator at the company BeiGene (Beijing) Co., Ltd. LJ is a Senior Scientist at the company BeiGene (Beijing) Co., Ltd. XL is the Vice President, Head of Biology at the company BeiGene (Beijing) Co., Ltd.

## Publisher’s note

All claims expressed in this article are solely those of the authors and do not necessarily represent those of their affiliated organizations, or those of the publisher, the editors and the reviewers. Any product that may be evaluated in this article, or claim that may be made by its manufacturer, is not guaranteed or endorsed by the publisher.
